# Interpreting scan data acquired from multiple scanners: A study with Alzheimer's disease

**DOI:** 10.1016/j.neuroimage.2007.09.066

**Published:** 2008-02-01

**Authors:** Cynthia M. Stonnington, Geoffrey Tan, Stefan Klöppel, Carlton Chu, Bogdan Draganski, Clifford R. Jack, Kewei Chen, John Ashburner, Richard S.J. Frackowiak

**Affiliations:** a Wellcome Trust Centre for Neuroimaging, Institute of Neurology, UCL, London, UK; b Department of Psychiatry and Psychology, Mayo Clinic, Scottsdale, AZ, USA; c Department of Neurology, Neurozentrum, University Clinic Freiburg, Freiburg, Germany; d Department of Radiology, Mayo Clinic, Rochester, MN, USA; e Banner Alzheimer's Institute, Banner Samaritan PET Center, Phoenix, AZ, USA; f Département d'études cognitives, Ecole Normale Supérieure, Paris, France; g Laboratory of Neuroimaging, IRCCS Santa Lucia, Rome, Italy

**Keywords:** Multi-scanner, Magnetic resonance imaging (MRI), Alzheimer's disease, Voxel based morphometry

## Abstract

Large, multi-site studies utilizing MRI-derived measures from multiple scanners present an opportunity to advance research by pooling data. On the other hand, it remains unclear whether or not the potential confound introduced by different scanners and upgrades will devalue the integrity of any results. Although there are studies of scanner differences for the purpose of calibration and quality control, the current literature is devoid of studies that describe the analysis of multi-scanner data with regard to the interaction of scanner(s) with effects of interest. We investigated a data-set of 136 subjects, 62 patients with mild to moderate Alzheimer's disease and 74 cognitively normal elderly controls, with MRI scans from one center that were acquired over 10 years with 6 different scanners and multiple upgrades over time. We used a whole-brain voxel-wise analysis to evaluate the effect of scanner, effect of disease, and the interaction of scanner and disease for the 6 different scanners. The effect of disease in patients showed the expected significant reduction of grey matter in the medial temporal lobe. Scanner differences were substantially less than the group differences and only significant in the thalamus. There was no significant interaction of scanner with disease group. We describe the rationale for concluding that our results were not confounded by scanner differences. Similar analyses in other multi-scanner data-sets could be used to justify the pooling of data when needed, such as in studies of rare disorders or in multi-center designs.

## Introduction

Techniques utilizing in vivo MRI-derived measures of brain tissue morphometrics show increasing promise for aiding clinicians in assessment and diagnosis of disease. Studies that use longitudinal and/or multi-site data-sets, such as the Alzheimer's Disease Neuroimaging Initiative (Mueller et al., 2005), have the potential to provide a wealth of information. The large numbers of subjects resulting from pooling multi-scanner data-sets has numerous advantages. It increases sensitivity thus allowing detection of subtle effects and enables the analysis of subgroups within a cohort. Additionally, pooling offers increased reliability and confidence about the size of effect by averaging out unforeseen confounds and hence a method for carrying out meta-analyses or analyses of rare subjects with orphan-diseases scanned at home rather than in distant centers. Any one study may have unforeseen bias which is lessened by pooling. However, one important confound of combining images gathered from different scanners is the potential for scanner effects to introduce systematic error, thus making the interpretation of results difficult. Partial volume effects and image intensity inhomogeneity can introduce error into automatic segmentation with any given scanner (Li et al., 2005). Noise of the electronics of the MRI system, subject-specific physiological noise and imaging gradient non-linearities also contribute to image intensity variability (Jovicich et al., 2006; Littmann et al., 2006). Furthermore, differences in subject positioning between sites add to the variability in distortion fields (Jovicich et al., 2006) and images can vary as a function of protocol differences between a baseline and a later scan or with drifts in instrument signal to noise over time (Preboske et al., 2006). The interaction of scanner differences with segmentation remains a particular concern and potential cause of varied measures of regional tissue volume in the brain (Ashburner and Friston, 2000). It is therefore vital to confirm that there is no important interaction between scanner and effect of interest and/or account for the effects of different scanners in a principled manner before pooling from different scanners can be recommended as a routine.

The use of phantoms to calibrate different scanners and account for scanner drift or upgrades is well established (Tofts, 1998; Van Haren et al., 2003; Schnack et al., 2004; Jovicich et al., 2006) and a useful way to guarantee quality. Studies using a small number of healthy subjects repeatedly scanned on different scanners or after upgrades are also recommended to test reliability (Tofts, 1998; Han et al., 2006; Jovicich et al., 2006). However, it is not clear that the extra calibration efforts can actually correct for the majority of these sources of variability (Jovicich et al., 2006), or are even necessary. Also, it has yet to be established how confidently the results from multi-scanner data-sets can be interpreted irrespective of up-front calibration.

Multi-scanner studies, though not common, are increasing. A longitudinal aging study by Raz and colleagues, one of the few which discussed the analysis of images derived from multiple scanners, concluded that using different scanners in 4 subjects did not affect measured intracranial volume with a manual tracing method (Raz et al., 2005). Likewise, manual hippocampal measurements performed on both 1.5 T and 3.0 T scanners in 8 healthy controls were not affected by field strength (Breillmann et al., 2001). We are not aware of any study that has described an automated analysis of a large, multi-scanner data-set with the aim of assessing whether scanner-associated biases are significant.

We set out to investigate scanner effects in an automatically preprocessed data-set of subjects with mild to moderate AD and cognitively normal elderly people whose MRI scans had been collected over a period of 10 years with multiple scanners and upgrades on a consistent platform and repeated calibrations. Even though the concern has been raised that segmentation errors are a particular problem in atrophic brains (Good et al., 2002), we did not find this to be an issue in our sample, possibly because the disease was early. We hypothesized that the changes due to AD pathology are large compared to scanner induced distortions and that there would be no interaction of scanner with case and/or control groups. In this paper, we report the results in our data-set and outline a method by which multi-scanner data-sets might be pooled with confidence.

## Methods and results

Our data-set included a total of 6 scanners and 136 subjects scanned over a 10-year period. None of the subjects were scanned more than once. All scans were done on the same platform, General Electric Signa 1.5 T scanners (slice thickness 1.6, matrix dimensions 256 × 192). There were minor variations in the TR, TE, and flip angle (see Table 1). Scanners also underwent upgrades over time. Importantly, all scanners were monitored with daily phantom quality checks which calibrated the gradients to within ± 1 mm over a 200-mm volume centered at iso-center, monitored signal to noise and radio frequency (RF) transmit gain. The major hardware elements (body resonance module gradient coil and birdcage head transmit–receive volume coil) were unchanged throughout time and across scanners, except that for the oldest scanner (scanner 2), the wiring in the resistive shim set was not cooled to super conducting temperatures, whereas for the other 5 scanners, the shim coils were inside the dewar and were cooled to the superconducting range.

We used voxel-based morphometry (VBM) to evaluate the interaction of scanner and grey matter segmented modulated images for the 6 different scanners. VBM has the advantage of assessing the whole brain and not being biased to one particular region or structure. It entails a voxel-wise comparison of local volume of grey matter between groups after the images are spatially normalized into the same space, segmented, modulated, and smoothed. Voxel-wise statistical parametric maps result from statistically thresholded contrasts after corrections for multiple comparisons (Ashburner and Friston, 2000) using false discovery rate (FDR) (Benjamini and Hochberg, 1995).

Images were visually inspected for artifacts or structural abnormalities unrelated to AD. They were firstly segmented into white (WM) and grey matter (GM) using SPM5 (Wellcome Trust Centre for Neuroimaging, Institute of Neurology, UCL, London UK – http://www.fil.ion.ucl.ac.uk/spm). Then, WM and GM segments were further normalized to a population template generated from the complete image set using a diffeomorphic registration algorithm. This non-linear warping technique minimizes structural variation between subjects (Ashburner, 2007). For comparison, we also repeated the analysis using the more widely used standard SPM5 segmentation code (Ashburner and Friston, 2005) instead of the diffeomorphic registration algorithm. Resolution before normalization was − .9, .9, 1.6 and after normalization was − 1.5, 1.5, 1.5 for the diffeomorphic registration algorithm and − 2.0, 2.0, 2.0 with the standard SPM5 procedure. A separate ‘modulation’ step (Ashburner and Friston, 2000) was used to ensure that the overall amount of each tissue class was not altered by the spatial normalization procedure. Modulation was performed by multiplying the warped tissue probability maps by the Jacobian determinant of the warp on a voxel-by-voxel basis, which represents the relative volume ratio before and after warping, thus allowing voxel intensities in the segmented grey matter map, together with the size of the voxels, to reflect regional volume and preserve total grey matter volume from before the warp. Modulated grey matter scans were smoothed using a 6-mm full-width at half-maximum Gaussian kernel.

**Fig. 1:**
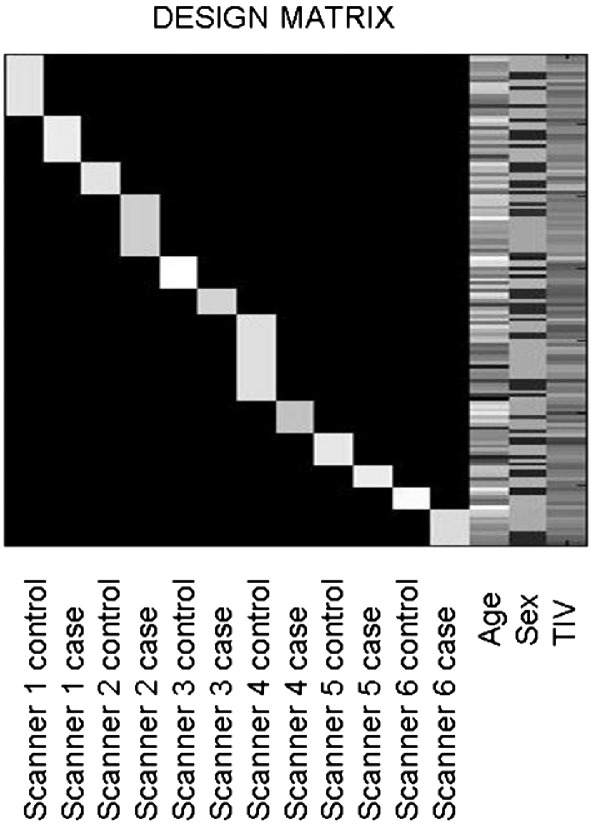
Design matrix. Six scanners are separated by patients and controls, i.e., scanner 1 normals, scanner 1 AD, scanner 2 normals, scanner 2 AD, etc. Nuisance covariates include age, sex, and total intracranial volume (TIV).

**Fig. 2:**
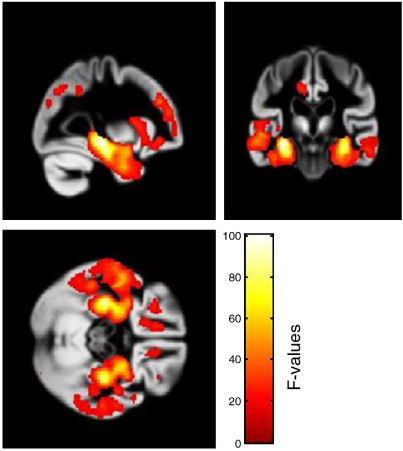
*F*-tests showing effect of disease group (AD vs. controls irrespective of scanner), FDR threshold of *p* < 0.001. Images are overlaid on a group average. Colour bar reflects the *F*-values.

**Fig. 3:**
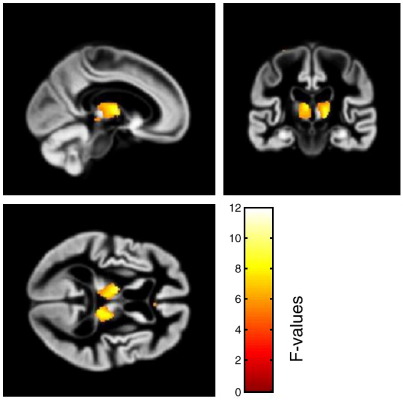
*F*-test showing effect of scanner (irrespective of cases or controls), FDR threshold of *p* < 0.05. Images are overlaid on a group average. Colour bar reflects the *F*-values.

The smoothed grey matter images were analyzed in a factorial design, with the 6 different scanners as one factor (SCANNER) with 6 levels and the presence of AD (GROUP) as the second factor with two levels (present and absent). Age, gender, and total intracranial volume were entered as covariates (Fig. 1). We performed *F*-tests correcting for multiple comparisons across the brain (FDR correction). The degrees of freedom was 121 for all comparisons. The effect of group revealed strong effects (*p* < 0.001) in the left medial temporal lobe (− 29, − 24, − 9 [*x*, *y*, *z*]; *F* = 100.34) and right medial temporal lobe (30, − 27, − 9 [*x*, *y*, *z*]; *F* = 91.44) (Fig. 2). The effect of scanner showed significant differences (*p* < 0.05) in the right (9, − 30, 0 [*x*, *y*, *z*]; *F* = 11.93) and left (− 9, − 11, 8 [*x*, *y*, *z*]; *F* = 9.69) thalami (Fig. 3), but this effect was less than the effect of group. *T*-tests contrasting each scanner against the others revealed the scanner effect in the thalamus was mostly due to the oldest scanner (scanner 2). One newer scanner (scanner 6) revealed an effect that did not survive FDR correction in the right thalamus (8, − 5, − 3 [*x*, *y*, *z*]; *T* = 4.03), whereas the oldest scanner contrasted with the others revealed a significant effect (*p* < 0.05) in the left thalamus (− 9, − 11, 6 [*x*, *y*, *z*]; *T* = 5.94). Despite the effect of scanner, there was no significant interaction of scanner with group, the highest *Z*-score being 3.82 with a corrected *p* value of 0.942. We performed *F*-tests for each possible combination of scanners and found no significant interaction with any of these groupings. When using the standard VBM procedure implemented in SPM5 for normalization and segmentation, the results of the various contrasts followed the same patterns as when using the diffeomorphic normalization/segmentation procedure. We failed to find an interaction of scanner with group; the highest *Z*-score was 3.78 with an FDR corrected p value of 0.954. The effect of scanner showed significant differences (*p* < 0.05) in the left thalamus (− 10, − 10, 6 [*x*, *y*, *z*]; *F* = 9.49) and the right thalamus (8, − 28, − 4 [*x*, *y*, *z*]; *F* = 9.41). The effect of group also showed strong effects (*p* < 0.001) in the left medial temporal lobe (− 26, − 14, − 20 [*x*, *y*, *z*]; *F* = 101.46) and right medial temporal lobe (26, − 8, − 18 [*x*, *y*, *z*]; *F* = 77.98).

**Fig. 4:**
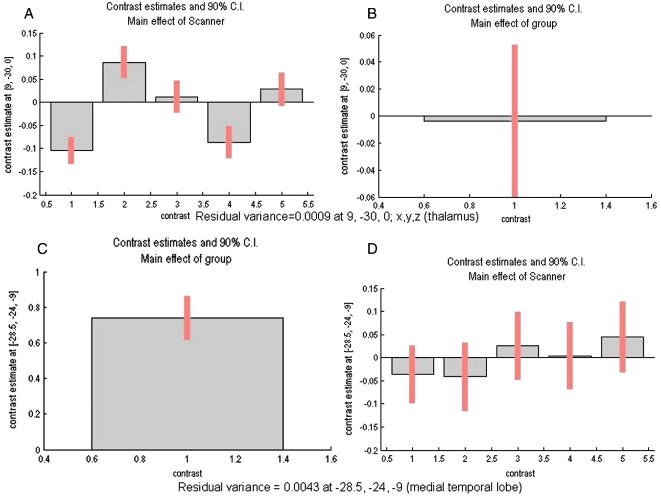
Contrast estimates and 90% confidence intervals for: (A) Main effect of scanner in thalamus at [9, − 30, 0; *x*, *y*, *z*] for contrasts of scanners 1 and 2; scanners 2 and 3; scanners 3 and 4; scanners 4 and 5; scanners 5 and 6. (B) Main effect of group in thalamus at [9, − 30, 0; *x*, *y*, *z*] for contrast of AD and controls. (C) Main effect of group in medial temporal lobe at [− 28.5, − 24, − 9; *x*, *y*, *z*] for contrast of AD and controls. (D) Main effect of scanner in medial temporal lobe at [− 28.5, − 24, − 9; *x*, *y*, *z*] for contrasts of scanners 1 and 2; scanners 2 and 3; scanners 3 and 4; scanners 4 and 5; scanners 5 and 6.

Importantly, we found no significant scanner effects in the medial temporal lobe cluster (− 29,− 24, − 9 [*x*, *y*, *z*]; *F* = 0.01) and the disease effect size in the thalamus was minimal compared to the effect size in the medial temporal lobe (9, − 30, 0 [*x*, *y*, *z*]; *F* = 1.06), suggesting minimal scanner effects in the areas that are most affected by AD and minimal disease effects in the areas showing scanner differences. Confidence intervals, which are reflective of the standard deviations, for the contrast estimates are shown in Fig. 4. At the voxel of greatest effect of group, the confidence interval is small relative to the effect size for the main effect of group. The opposite is true for the effect of scanner at the area of greatest disease, which is further evidence of lack of effect. For the main effect of scanner, confidence intervals are similar between the different scanner contrasts (see Fig. 4A), indirectly suggestive of relatively little variance across scanners.

Because there were 4 software upgrades, we also analyzed the interaction of software version and disease. Using the same basic design matrix as described for the interaction with scanners, this time the contrasts included cases and controls from each software version, covaried with age, gender and intracranial volume. As with the effect of scanner, there was no significant interaction of software version with group, the highest *Z*-score being 3.65 with a corrected *p* value of 0.880.

## Discussion

In our data-set, we found the effect of disease to be substantially larger than the effect of scanner and failed to find a significant interaction of disease with scanner or software upgrades. In general, the effect of disease in AD is liable to be larger than the effect of scanner, which is supported by our result of no important interaction between scanner and effect of interest. Ideally, further studies with an even larger data set to better calculate the effect sizes and quantify the distance between the scanner effect cluster and group effect cluster could be done to validate our findings. However, comparison of the magnitude of the scanner effect versus disease effect in the medial temporal lobe cluster in our data-set demonstrates that scanner differences had minimal effects in the areas that are important in the study of AD. Furthermore, even though there are likely to be some differences among data from different scanners, our experiments were explicitly designed to detect scanner-related differences. By modeling appropriate confounds in the design matrix, it appears possible to remove these small effects. We were able to model this interaction easily because relatively homogeneous cases and controls were scanned in each machine. We are unable to say whether lesser or more subtle and distributed changes would be as resistant to scanner effects.

Though we did not detect a significant interaction of scanner with disease, we cannot be absolutely certain it is indeed due to the absence of such effect. The lack of significance may be a reflection of the lack of statistical power, e.g., insufficient number of scans. Other causes such as a high average residual variance or residual variance inhomogeneities could also under-power the detection of the effects. However, the variance inhomogeneity was considered in our analysis by assuming unequal variance for the different levels of each of the two factors in our full factorial design (the two factors are the scanner and group). In a post-hoc manner, we explored the residual variance across scanners to assess whether that explained the lack of sensitivity in the results. In the area of greatest disease effect, the variance was low and did not reflect significant inhomogeneity, but in the area of greatest scanner effect there was lower average residual variance and more variability. Though our tests for variance inhomogeneity across the 6 scanners was not voxel-by-voxel over the whole brain volume, our findings seem to support that the variance inhomogeneity is location-dependent and should be accounted for when analyzing data acquired from different scanners as we attempted to do in our analysis.

The greatest effect of scanner was in the thalamus. The effect of the thalamus was largely driven by the scanner with the resistive shim set that was not cooled to superconducting temperatures, which suggests an impact of such hardware differences on thalamic segmentation. The composition of the thalamus is an issue of debate as it is not completely grey matter receiving numerous white matter tracts from other parts of the brain. Additionally, the grey matter intensity value of the thalamus is different from that of cortical grey matter. The intrinsically poor intensity contrast in the thalamus renders it susceptible to small differences in image contrast due to scanner differences. There is also less variability in this part of the brain, so tests will be more sensitive to such differences. These factors may contribute to the difficulty of accurate segmentation of the thalamus in addition to scanner effects.

The relatively small effect of scanner is potentially attributable to quality control measures during data acquisition and/or the robustness of the segmentation method. The SPM5 algorithm, by using spatial information together with intensity information, should be more robust to such differences than a segmentation algorithm that is purely intensity-based. However, this study did not directly investigate which forms of preprocessing are most affected by scanner parameters. It is important to note, though, that the effect was not due to the diffeomorphic normalization procedure, since we obtained similar patterns to those with the standard SPM5 normalization. Presumably, the use of daily scanner calibrations allows for a relatively constant intensity contrast between grey and white matter voxels between scans. Routine procedures designed to minimize image intensity differences over time are useful, particularly since it has been demonstrated that several common scan functions can potentially introduce measurement errors as high as 100% (i.e., much greater than the disease effect of AD), without appropriate quality control measures in place (Preboske et al., 2006). Even though it is common experience that current generation scanners are remarkably stable over time, they do still drift in a way that is correctable with judicious calibration. Though we collected no data to determine the frequency and level of sophistication necessary, basic calibration akin to the current standard in most major centers is likely sufficient. Furthermore, while it can be useful to collect data on inter-subject variability by scanning the same subjects in different scanners, such efforts would not directly answer the question of whether data from different subjects on different scanners can be pooled.

The unified segmentation method of SPM5 (Ashburner and Friston, 2005) produces an estimate of the tissue class intensities from a fitting of spatial priors to the image, which allows for differences in image intensities between scans. Additionally, any image intensity differences due to subject to subject variation in coupling of the RF coil to the head that may be introduced by different head shapes and sizes are likely to be well-accounted for by bias corrections in the unified segmentation algorithm.

As long as provision for different scanners and/or upgrades is made within an analysis, the effect of scanner regardless of magnitude is not likely to devalue the integrity of results. However, if there is a true physical interaction of the biological effect of interest with the method of measurement, perfect calibration or even using a single scanner would not prevent bias. On the other hand, any unusually large effect from one scanner would be attenuated by the totality of scans from different scanners that make up the template, which averages the different scanner effects for normalization.

To date, methodological differences between individual studies have prevented comprehensive pooling of data in meta-analyses of AD MRI studies (Zakzanis et al., 2003; Whitwell and Jack, 2005; Wahlund et al., 2005). To the extent that data pooling was possible, the meta-analysis compared regional volume between disease groups (Zakzanis et al., 2003). Although there may be concern about error introduced by the variability inherent in preprocessing many individuals across studies, automated VBM methods successfully overcome the problems to combine information from multiple scans and studies. VBM meta-analyses of pooled scans permit the usual range of analyses beyond simple categorical comparisons (e.g., regression, age interaction, subgroup, nonlinear, etc.).

Our results suggest that for Alzheimer's disease, particularly if the imaging platform remains constant, variations attributable to individual scanners and upgrades may have negligible effects on segmented grey matter images. We expect, though do not yet have the data, that the approach is robust enough to accommodate using both 1.5 T and 3 T scans in the same meta-analysis. A principled approach to test the validity of a meta-analysis is to carry out an *a priori* interaction analysis of scanner by biological effect to be studied to exclude scanner-specific compromising effects. Additional comfort can be gained by finding that the biological effect is of greater magnitude than any effect of scanner. An alternative approach is to model each scanner's effects separately, thereby permitting a principled meta-analysis on pooled data. That data can be pooled from different scanners without corroding the integrity of results is reassuring for large multi-site studies.

## Figures and Tables

**Table 1:** Scanner and subject demographics (^⁎^)

Scanner	Group	MMSE^a^ (range)	Age (range)	Sex (F/M)	*n*	TR	TE	Flip angle (deg)
1	Controls	28.6 (26–30)	75.1 (61–85)	4/13	17	17.5–27	6–10	25, 45
Patients	23.2 (17–27)	75.8 (63–87)	5/8	13	23–27	6–10	25, 45
2	Controls	29 (26–30)	78.6 (68–87)	6/3	9	24	9	45
Patients	22.6 (15–29)	78.5 (68–92)	4/13	17	24	9	45
3	Controls	28.7 (26–30)	80.1 (70–90)	3/6	9	23–25	9–10	25, 45
Patients	22.4 (15–27)	76.4 (61–88)	6/1	7	23–27	9–10	25, 45
4	Controls	29.0 (27–30)	72.1 (50–86)	7/17	24	23,25	6–10	25, 45
Patients	21.9 (18–27)	80.2 (54–91)	2/7	9	23	6–10	25
5	Controls	29.3 (28–30)	70.4 (57–79)	4/5	9	22,23	9.8,10	25
Patients	22 (17–29)	77.3 (66–83)	2/4	6	17.7–27	6–10	25, 45
6	Controls	27.7 (26–30)	78.7 (68–91)	2/4	6	17.6–27	6–10	25, 45
Patients	20.4 (17–24)	76.3 (71–85)	5/5	10	21–27	6–10	25, 45

a MMSE = Mini-Mental State Exam.
